# IgG Seroconversion and Pathophysiology in Severe Acute Respiratory Syndrome Coronavirus 2 Infection

**DOI:** 10.3201/eid2701.203074

**Published:** 2021-01

**Authors:** Henry M. Staines, Daniela E. Kirwan, David J. Clark, Emily R. Adams, Yolanda Augustin, Rachel L. Byrne, Michael Cocozza, Ana I. Cubas-Atienzar, Luis E. Cuevas, Martina Cusinato, Benedict M.O. Davies, Mark Davis, Paul Davis, Annelyse Duvoix, Nicholas M. Eckersley, Daniel Forton, Alice J. Fraser, Gala Garrod, Linda Hadcocks, Qinxue Hu, Michael Johnson, Grant A. Kay, Kesja Klekotko, Zawditu Lewis, Derek C. Macallan, Josephine Mensah-Kane, Stefanie Menzies, Irene Monahan, Catherine M. Moore, Gerhard Nebe-von-Caron, Sophie I. Owen, Chris Sainter, Amadou A. Sall, James Schouten, Christopher T. Williams, John Wilkins, Kevin Woolston, Joseph R.A. Fitchett, Sanjeev Krishna, Tim Planche

**Affiliations:** St. George’s, University of London, London, UK (H.M. Staines, D.E. Kirwan, D.J. Clark, Y. Augustin, M. Cuisinato, B.M.O. Davies, N.M. Eckersley, D. Forton, L. Hadcocks, Q. Hu, D.C. Macallan, I. Monahan, C.M. Moore, S. Krishna, T. Planche);; Liverpool School of Tropical Medicine, Liverpool, UK (E.R. Adams, R.L. Byrne, A.I. Cubas-Atienzar, L.E. Cuevas, A.J. Fraser, G. Garrod, G.A. Kay, S. Menzies, S.I. Owen, C.T. Williams);; Mologic, Thurleigh, UK (M. Cocozza, M. Davis, P. Davis, A. Duvoix, M. Johnson, K. Klekotko, Z. Lewis, J. Mensah-Kane, G. Nebe-von-Caron, C. Sainter, J. Schouten, J. Wilkins, K. Woolston, J.R.A. Fitchett);; St. George’s University Hospitals National Health Services Foundation Trust, London (D. Forton, S. Krishna, T. Planche);; Institut Pasteur, Dakar, Senegal (A.A. Sall);; Universitätsklinikum Tübingen, Tübingen, Germany (S. Krishna);; Centre de Recherches Médicales de Lambaréné, Lambaréné, Gabon (S. Krishna)

**Keywords:** SARS-CoV-2, COVID-19, diagnostics, immunology, antibody responses, respiratory infections, severe acute respiratory syndrome coronavirus 2, 2019 novel coronavirus disease, coronavirus disease, zoonoses, viruses, coronavirus, antibodies, serology, United Kingdom

## Abstract

We investigated the dynamics of seroconversion in severe acute respiratory syndrome coronavirus 2 (SARS-CoV-2) infection. During March 29–May 22, 2020, we collected serum samples and associated clinical data from 177 persons in London, UK, who had SARS-CoV-2 infection. We measured IgG against SARS-CoV-2 and compared antibody levels with patient outcomes, demographic information, and laboratory characteristics. We found that 2.0%–8.5% of persons did not seroconvert 3–6 weeks after infection. Persons who seroconverted were older, were more likely to have concurrent conditions, and had higher levels of inflammatory markers. Non-White persons had higher antibody concentrations than those who identified as White; these concentrations did not decline during follow-up. Serologic assay results correlated with disease outcome, race, and other risk factors for severe SARS-CoV-2 infection. Serologic assays can be used in surveillance to clarify the duration and protective nature of humoral responses to SARS-CoV-2 infection.

Severe acute respiratory syndrome coronavirus 2 (SARS-CoV-2) is a betacoronavirus that causes coronavirus disease (COVID-19), a respiratory infection with systemic involvement and an estimated 1% death rate (*1*). COVID-19 was first documented in Wuhan, China (*2*), at the end of 2019. The outbreak quickly transformed into a pandemic. Countries have tried to manage the pandemic by implementing different strategic interventions with varying levels of success (*3*). Experts agree that diagnostic tests, and the subsequent interventions they generate, are essential to controlling SARS-CoV-2 transmission.

Reverse transcription PCR (RT-PCR) relies on RNA sequencing rather than viral proteins, enabling researchers to develop assays shortly after the viral sequence is identified. Because of this advantage, RT-PCR quickly became a common testing method for COVID-19 (*4*). However, the urgent global scale-up of nucleic acid amplification testing, including PCR, exposed supply chain vulnerabilities, such as shortages of swabs and reagents. Diagnostic tests remain unaffordable in many developing countries, limiting national containment strategies.

Serologic assays for viral infections can contribute to vaccine development, diagnostic deployment, and prescription of new therapeutics. They might also offer insight into pathophysiological aspects of COVID-19. We used ELISA (Mologic Ltd., https://mologic.co.uk) for COVID-19 to characterize the serologic response in SARS-CoV-2 infection. These tests were designed for affordability and accuracy, enabling access and manufacture in low- and middle-income countries. We used these tests on serum samples from persons with confirmed SARS-CoV-2 infection in London, UK, to identify demographic and clinical variables that might influence antibody responses.

## Methods

### Ethics

Development of the SARS-CoV-2 IgG ELISA is available elsewhere (E.R. Adams, unpub. data, https://www.medrxiv.org/content/10.1101/2020.04.29.20082099v1). We analyzed antibody dynamics using anonymized excess diagnostic material from patients with PCR-confirmed SARS-CoV-2 infection. The study was sponsored by St. George’s Hospital National Health Services Foundation Trust (London) and has Institutional Review Board ethics approval (Development and Assessment of Rapid Testing for SARS-CoV-2 outbreak study; Integrated Research Application System project ID: 282104; Research Ethics Committee reference: 20/SC/0171). The trial is registered at ClinicalTrials.gov under NCT04351646.

### Reference RT-PCR

Staff at St. George’s Hospital used Sigma Virocult (MWE, https://www.mwe.co.uk) to collect nose and throat swab samples from patients with SARS-CoV-2 infection; we prepared the samples with RNA extraction kits (Roche Molecular Systems Inc., https://www.lifescience.roche.com). We confirmed infection with the RealStar SARS-CoV-2 RT-PCR Kit selective for the S and E genes (Altona Diagnostics GmbH, https://www.altona-diagnostics.com) or cobas SARS-CoV-2 Test selective for the E gene and open reading frames 1ab (Roche Molecular Systems, Inc.).

### Clinical Samples

South West London Pathology (London) provides microbial diagnostic testing for the region, including St. George’s Hospital, a tertiary teaching hospital. We obtained excess diagnostic material from South West London Pathology in the form of serum samples from patients with RT-PCR confirmed SARS-CoV-2 infection. The serum samples were anonymized and stored at 4°C for <2 weeks. Patients were sampled longitudinally to assess antibody dynamics; the data comprised >30 samples per day. If samples became unavailable from 1 patient (i.e., the patient was discharged or died), we added a new patient to the cohort. Excess diagnostic material was collected from 177 persons during March 29–May 22, 2020. The study population consisted of 9.9% (177/1,785) of persons (patients and staff) who tested positive for SARS-CoV-2 infection at South West London Pathology during this period.

### Participants and Clinical Data

We obtained data from patients’ electronic medical records. We coded outcomes (as of May 22) as hospital admission, intensive care unit stay, death, or discharge. We recorded the length of hospital stay of patients who were discharged or died. We considered peaks of inflammatory markers (e.g., C-reactive protein [CRP]) to be the highest values recorded from 5 days before the first positive swab sample through the end of the study. We obtained blood values at the time of diagnosis (within 3 days after the first positive swab sample was taken).

### ELISA for SARS-CoV-2 IgG

We used the COVID-19 IgG ELISA developed by Mologic Ltd. and manufactured by Omega (Omega Diagnostics Group PLC, http://www.omegadiagnostics.com), according to the manufacturer’s instructions (Appendix 1). The assay contained the spike and nucleoprotein antigens of SARS-CoV-2. Between plate coefficients of variation were 21.0% (lower cutoff) and 16.5% (positive control; n = 16). Higher ambient temperatures in the laboratory resulted in higher optical density readings (Appendix 1).

### Statistical Analyses

We cross-checked and normalized raw ELISA data to enable comparison (Appendix 1). We also resolved manual handling errors (Appendix 1). We applied 2-tailed parametric and nonparametric tests as appropriate, using PRISM version 8.0 (https://www.graphpad.com) for data analysis and display. We conducted a 1-way analysis of variance to compare the effects of race and demographic information on patient outcomes. We used multivariate linear regression to determine the relationship between mean normalized optical density (NOD) and age, sex, peak CRP, number of concurrent conditions, respiratory symptoms, and race.

### Patient and Public Involvement

We acknowledge the importance of patient and public involvement in clinical studies. However, because of the rapid progression of COVID-19 and the challenges of lockdown in the United Kingdom, we did not have sufficient time to involve patients and members of the public in the development, implementation, or interpretation of this study.

## Results

We studied 177 patients who provided 645 distinct excess diagnostic material samples (Table 1). Patients were from diverse ethnic backgrounds (34% White, 35% non-White, 31% unreported; Appendix 1), and the median age was 64 years (interquartile range [IQR] 52–77 years). Fifty-seven percent were male, and 73% had >1 concurrent condition. Nineteen percent were asymptomatic and did not report respiratory symptoms at admission; these patients tested positive for SARS-CoV-2 infection while receiving treatment for other conditions. Among the 143 symptomatic patients, the median time from symptom onset to testing was 6 days (IQR 3–9 days). Of the 177 patients, 166 (94%) were hospitalized, 7 (4%) were staff, and 4 (2%) were outpatients. Of the hospitalized patients, 44 (27%) died (median time to death was 19.1 days [IQR 14.8–24.8 days]), 108 (65%) were discharged (median length of stay was 19.3 days [IQR 10.6–31.1 days]), and 14 (8%) remained hospitalized at the end of the study. Sixty-three (38%) patients were admitted to intensive care during the study.

**Table 1:** Demographic and clinical characteristics of patients with severe acute respiratory syndrome coronavirus 2 infection, United Kingdom, 2020*

Characteristics	Results
Median age, y (IQR)	64 (52–77)
Median body mass index (IQR)†	25.4 (21.9–30.5)
Sex	
M	100 (56.5)
F	77 (43.5)
Race	
White	60 (33.9)
Non-White	61 (34.5)
Other/not known	56 (31.6)
Concurrent conditions	
0	47 (26.6)
1	52 (29.4)
2	50 (28.2)
>3	28 (15.8)
Symptoms	
Symptomatic	143 (80.8)
Median days from symptom onset to PCR (IQR)	6 (3–9)
Diagnostic site	
Emergency department	90 (50.8)
Outpatient	12 (6.8)
Ward	54 (30.5)
Intensive care unit	14 (7.9)
Occupational health staff	7 (4.0)
Treatment location	
Occupational health staff	7 (4.0)
Outpatient	4 (2.3)
Hospital	166 (93.8)
Admitted to intensive care unit	63 (38.0)
Outcomes	
Never hospitalized	11 (6.2)
Discharged	108 (61.0)
Median length of stay, d (IQR)	19.3 (10.6–31.1)
Death	44 (24.9)
Median length of stay, d (IQR)	19.1 (14.8–24.8)
Death and/or ICU admission	80 (45.2)
Still in hospital‡	14 (7.9)

*Values are no. (%) except as indicated. ICU, intensive care unit; IQR, interquartile range. †Height unavailable for 13 patients. ‡As of May 22, 2020.

**Figure 1:**
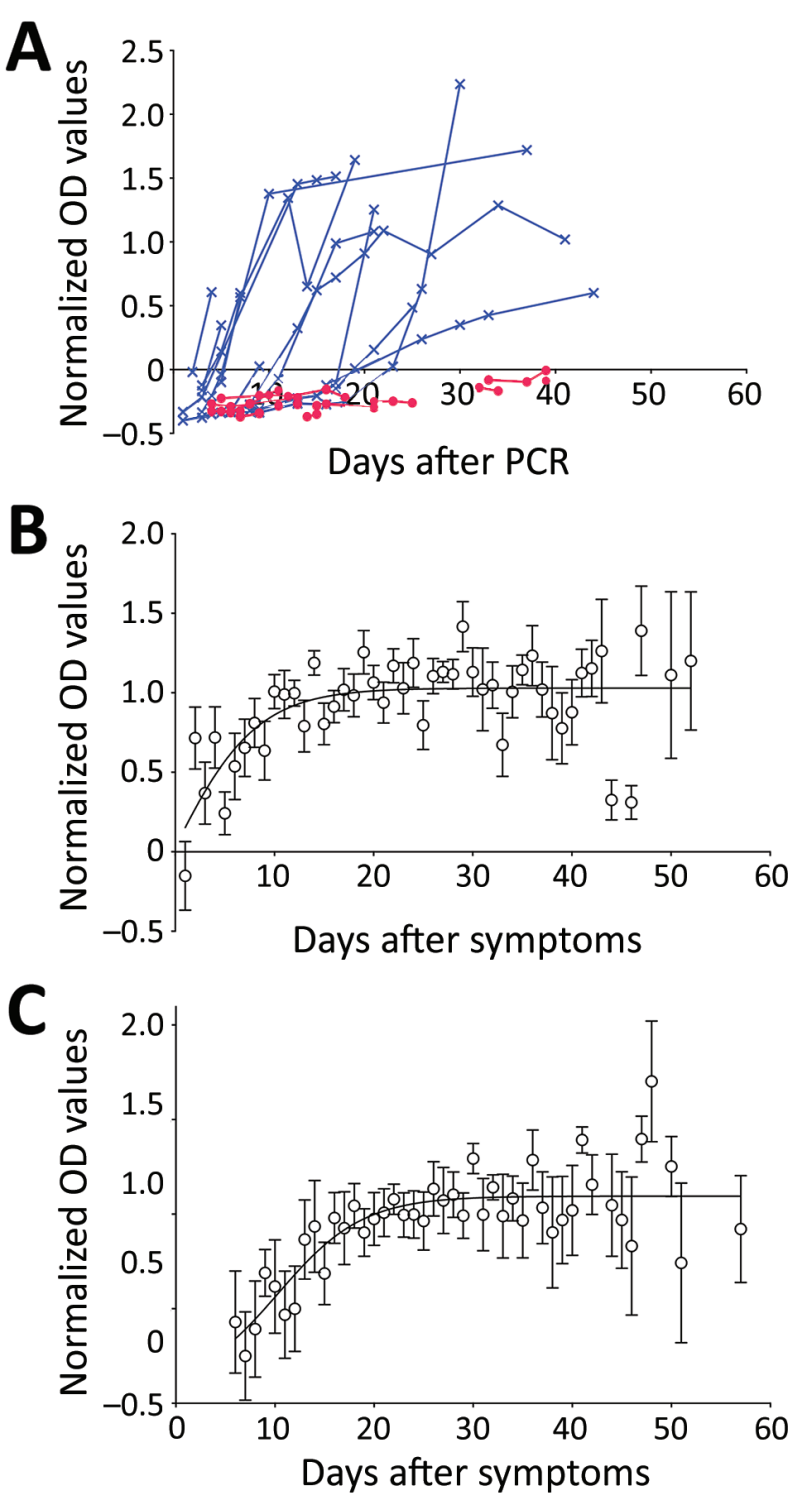
Antibody dynamics in patients with severe acute respiratory syndrome coronavirus 2, United Kingdom, 2020. A) NOD by days after first positive PCR result. Blue indicates seroconverting patients; red indicates nonseroconverting patients. B) Mean (± SEM) NODs (>3 samples per time point; n = 48) by days after first positive PCR result for those who seroconverted. A 4-parameter sigmoidal unconstrained model is shown (r^2^ = 0.45). C) Mean (± SEM) NODs (>3 per time point; n = 45) by days after symptom onset for patients who seroconverted. A 4-parameter sigmoidal unconstrained model is shown (r^2^ = 0.63). NOD, normalized optical density.

We normalized optical densities proportional to levels of SARS-CoV-2 IgG (Figure 1, panel A). Of the 177 patients, 149 (84% [95% CI 78%–89%]) had already seroconverted at the time of the first serologic test, 13 (7.3% [95% CI 4.3%–12.1%]) seroconverted after the first serologic test, and 15 (8.5 % [95% CI 5.2–13.5%]) did not seroconvert during the entire follow-up period (Appendix 1). Of the 15 patients who did not seroconvert, samples from beyond day 20 were available for 4 patients (26%); we did not detect IgG in these samples. This finding suggests that 2.0%–8.5% of patients might not develop detectable IgG against SARS-CoV-2.

We plotted NODs by time after a patient’s first positive swab sample (Figure 1, panel B) and after symptom onset (Figure 1, panel C). NODs plateaued »12 days after PCR and »19 days after symptom onset; this time difference is consistent with the median time of 6 days between symptom onset and PCR. After seroconversion, mean NODs remained stable over the course of the study (up to »60 days after symptom onset).

We assessed whether the rate of seroconversion was associated with patient age (<70 or >70 years), sex, or respiratory symptoms. None of these variables were discernably associated with seroconversion rates (Appendix 1). NOD IgG levels were not associated with sex or the presence of respiratory symptoms. (Appendix 1).

Patients of non-White race had higher mean NODs than those of White race (1.06 vs. 0.85; *F* = 1.61, df = 119; p = 0.04 by unpaired Student *t*-test) (Appendix 1). No other differences were associated with race. We used a multivariate analysis to identify variables independently associated with NODs; the mean NOD was associated only with age, peak CRP, and race. Although age, sex, peak CRP, number of concurrent conditions, respiratory symptoms, and race were associated with patient outcome in the univariate analysis, only peak CRP was associated with poor outcome in the multivariate analysis (Appendix 1).

Persons who seroconverted were older than those who did not (median age 65.5 vs. 41.0 years; p<0.01 by Mann-Whitney test) and more likely to have >1 concurrent condition (124/130 vs. 38/47; p<0.01 by Fisher exact test). History of hypertension was associated with a higher probability of seroconversion (74/75 persons with hypertension vs. 88/102 persons without hypertension; p<0.01 by Fisher exact test). Body mass index was higher among the group who seroconverted (25.7 vs. 21.2; p = 0.03 by Mann-Whitney test).

**Figure 2:**
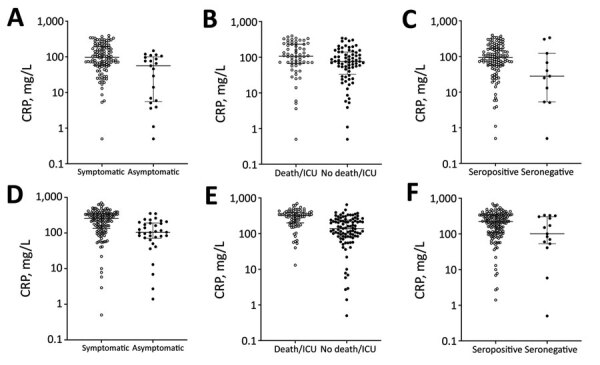
Relationships between CRP levels, symptoms, outcomes, and NODs of patients with severe acute respiratory syndrome coronavirus 2, United Kingdom, 2020. A–C) CRPs at diagnosis for A) 113 symptomatic (open circles) and 21 asymptomatic (closed circles) patients (CRP 97 vs. 56; p<0.01); B) 62 patients admitted to intensive care and/or who died (open circles) and 72 who were not admitted to intensive care (closed circles) (CRP 107 vs. 75.5; p = 0.01); C) 123 patients who seroconverted (open circles) and 11 who did not (closed circles) (CRP 93 vs. 28; p = 0.04). D–F) Peak CRPs corresponding to the populations in A–C: D) 255 (n = 142) vs. 104 (n = 34) (p<0.01); E) 322 (n = 80) vs. 137.5 (n = 96) (p<0.01); F) 224 (n = 161) vs. 101 (n = 15) (p = 0.03). Statistical significance calculated using Mann-Whitney test for CRPs (mg/L). CRP, C-reactive protein; NOD, normalized optical density.

Unlike other markers of inflammation, CRP is routinely measured in patients with COVID-19. Rising CRP levels are indicators of a poor prognosis (if other causes are excluded), and are associated with cytokine release syndrome (*5*; Y. Woo, unpub. data, https://osf.io/mxsvw). CRP levels were significantly higher in patients with respiratory symptoms at diagnosis than in those without symptoms (Figure 2, panel A). Patients who died or required intensive care during the study period had higher CRP levels than patients who did not die or require intensive care (Figure 2, panel B). Patients who did not seroconvert had lower CRP levels than those who did (Figure 2, panel C). Peak CRP levels had more pronounced associations with outcomes and seroconversion than did CRP levels at the time of the first positive swab sample result (Figure 2, panels D–F). CRP levels peaked a median of 12 days (IQR 8–17 days) after symptom onset and 4 days (IQR 1–11 days) after the first positive PCR result. Other inflammatory markers, such as peak D-dimer, fibrinogen, and ferritin, were also higher in patients with respiratory symptoms at diagnosis. However, these data were available for fewer patients (Table 2; Appendix 1).

**Table 2:** Selected laboratory values of patients with severe acute respiratory syndrome coronavirus 2 infection, United Kingdom, 2020*

Variable (reference range)	At diagnosis			Peak	
	No.	Median (IQR)		No.	Median (IQR)
C-reactive protein (0–5 mg/L)	134	86 (52.5–164)		176	215.5 (103–334)
Nadir lymphocytes count (1.1–4.0 × 10^9^/L)	134	0.9 (0.6–1.4)		177	0.6 (0.4–0.8)
Ferritin (30–400 μg/L)	42	1,084 (630–1,721)		89	1,335 (846–2,758)
Fibrinogen (1.6–4.8 g/L)	133	5.5 (4.2–6.7)		166	7.0 (5.5–9.15)
D-dimer (21–300 ng/mL)	64	704 (395–1,079)		111	1,905 (498–4,095)
Lactate dehydrogenase (0–250 U/L)	47	475 (280–597.5)		87	490 (344.5–704)

*IQR, interquartile range.

## Discussion

Our results illustrate serologic responses over the course of SARS-CoV-2 infection. Serologic tests can enhance diagnostic capability, especially during later infection (*4*,*6*,*7*), when viral loads might decrease. Serologic testing might also inform surveillance, seroepidemiologic studies, and contact tracing. Our study shows that a substantial proportion of COVID-19 patients require 3–6 weeks to generate antibodies. Furthermore, 2.0%–8.5% of patients do not have detectable antibodies within 60 days after infection. Most research on antibody dynamics came from China during the early stages of the pandemic (*4*,*6*,*8*). Here, we describe variables that influence IgG dynamics in SARS-CoV-2 infections in diverse populations.

The performance metrics of this ELISA (Appendix 2) are comparable to other validated assays. This first-generation ELISA might confirm infection in patients without a virologic diagnosis. We applied this test to study an ethnically and clinically diverse population. In most persons who seroconverted, the conversion was relatively rapid; NODs remained stable for weeks after infection (Figure 1). The probability of seroconversion was associated with increased age and concurrent conditions such as hypertension and increased body mass index. Higher NODs were associated with non-White race, admission to hospital, and higher peaks for inflammatory markers, such as CRP. Higher antibody titers are associated with clinical severity (i.e., death or admission to intensive care during study) in our cohort, in agreement with findings from other studies (*4*).

CRP is a sensitive marker of elevated proinflammatory cytokines, including interleukin 6. These cytokines might play a central role in cytokine release syndrome, which is associated with increased risk for death (Y. Woo, unpub. data, https://osf.io/mxsvw). Interventions such as tocilizumab, an interleukin-6 receptor antibody, interrupt the proinflammatory cascade. Such interventions might limit disease progression and reduce risk for death (*9*; E. Baker, unpub. data, https://osf.io/d2nh8); they are being studied in several randomized clinical trials. In our study, a small proportion of patients did not seroconvert within 20 days after testing positive for SARS-CoV-2 infection. Several mechanisms might explain this finding. First, these patients might never seroconvert. Second, their immune responses might be confined to other antigens or mediated through T cells. Another probable explanation is that some relatively mild infections might be restricted to the mucosal cells of the respiratory tract, where antibody responses are dominated by the secretory immune system. In this scenario, the systemic immune system might produce little or no IgG.

The association of higher NODs with elevated CRPs could indicate several potential pathways. For example, antibody responses might be closely related to cytokine response syndrome, which in turn is associated with more severe disease and death. Alternatively, elevated CRPs might indicate a more pronounced innate immune response in persons already at risk for severe disease and death. This heightened innate response might be associated with a higher viral load (potentially caused by enhanced viral replication mechanisms) and genetic interactions that influence innate inflammatory pathways. Therefore, a higher viral load might lead to higher NODs for antibodies in the acquired immune response pathways. Small trials on the potential therapeutic benefits of interventions using passive antibody transfer (*10*) suggest that the heightened innate response hypothesis is more probable (*11*). Higher antibody responses are also associated with higher doses of a nonreplicating Ad5-vectored vaccine for SARS-CoV-2 (*12*).

Limitations of PCR include difficulties with sampling; different sample types and techniques yield varying results. Furthermore, PCR demonstrates diminishing diagnostic yield for COVID-19 as respiratory viral loads fall and symptoms subside (*8*,*13*). It might also produce false positives caused by lingering viral nucleic acid, which is not infective yet can persist for weeks after infection. Contamination could also occur during sample handling; because PCR requires amplification steps, this assay has heightened risk for contamination. Serologic testing, and the ability to detect viral antigens, may increase diagnostic accuracy for COVID-19. Our findings support early studies suggesting that physicians should consider these diagnostic modalities in conjunction, especially when a patient has negative PCR results but has symptoms of COVID-19 (*6*). Many COVID-19 patients experience a delay in care, a trend that emphasizes the importance of containment strategies that encourage isolation.

One limitation of our study is that it is based mainly on hospitalized patients, of whom 1 in 5 did not have COVID-19 symptoms. Further studies should document antibody dynamics of patients with less severe infections, such as healthcare workers (*14*), and patients with low viral loads at the time of consultation. Our findings will complement the large cross-sectional and longitudinal serologic surveys, especially as high-quality tests become more widely available. NODs were within a limited dynamic range (we could not conduct dilution studies because of small sample volumes) but nevertheless associated with clinically relevant features of COVID-19. Prospective studies are assessing the relationships between viral loads and serologic responses in patients. Regular and long-term serologic assays will be essential to monitoring the duration of the humoral response and its protective role against SARS-CoV-2.

When interpreting serologic assays of COVID-19 patients, physicians should consider factors that can influence the probability of seroconversion. Our study elucidates some of these factors. We found that less severe infections and younger age were associated with reduced probability of seroconversion. Risk factors for more severe disease, such as non-White race, increased age, and hypertension, are also associated with increased inflammatory responses, higher normalized antibody titers, and probability of seroconversion.

Appendix 1 Additional information on methods, results, and analysis of IgG seroconversion and pathophysiology during COVID-19 infection.

Appendix 2 Comparison of commercially-available, independently validated ELISA platforms for severe acute respiratory syndrome coronavirus 2 IgG.
