# Supplementary material for: IgG Seroconversion and Pathophysiology in Severe Acute Respiratory Syndrome Coronavirus 2 Infection
Source: Emerg Infect Dis. 2021 Jan;27(1):85–91. doi: 10.3201/eid2701.203074 (PMC7774532; doi:10.3201/eid2701.203074)
Supplement: Appendix 1 — Additional information on methods, results, and analysis of IgG seroconversion and pathophysiology during COVID-19 infection. [file 20-3074-Techapp-s1.pdf]

Article DOI: https://doi.org/10.3201/eid2701.203074

IgG Seroconversion and Pathophysiology in Severe Acute Respiratory Syndrome Coronavirus 2 Infection

Appendix 1

Methods

ELISA

Serum samples were diluted 1 in 200 in dilution solution (10 mM Tris buffered saline, pH 7.2 with antimicrobial agents) and 100 μL loaded per well, in duplicate (although an initial plate was loaded in triplicate), onto a 96-well microtiter plate coated with the spike (S) and nucleoprotein (NP) antigens of SARS-CoV-2 (an early generation assay used NP, S1 and S2 antigens and the final version used only NP and S2 antigens, with S1 being removed). Six wells contained controls: negative (diluent solution), negative cutoff (purified human IgG in 10 mM Tris buffered saline) and positive (purified human IgG in 10 mM Tris buffered saline), in duplicates. The plate was incubated at room temperature for 30 min before 3 washes with wash buffer (10 mM Tris-buffered saline with detergent, pH 7.2). Following the wash step, 100 μL of conjugate solution (IgG against humans, conjugated to horseradish peroxidase with protein stabilization and antimicrobial agents) was added to every well and the plate incubated for 30 min at room temperature before 4 washes with wash buffer. Indicator/substrate solution (3,3′,5,5′-tetramethylbenzidine, TMB, with H_2O_2; 100 μL) was added to every well and the plate incubated at room temperature for 10 min before addition of 100 μL stop solution (0.25 M H_2SO_4) per well. The plate was read with a spectrometer at 450 nm within 10 min of stop solution addition. Wells with an optical density of 10% greater than the negative cutoff value were regarded as positive for antibodies to SARS-CoV-2 antigen.

Normalization

For each plate the mean cutoff OD value plus 10% (lower cutoff) was subtracted from each mean patient sample OD value (thus samples with negative values were considered

negative and samples with positive values were considered positive for SARS-CoV-2 antibodies). To normalize, the resulting value was divided by the mean positive control OD value. Any assayed duplicates (serum samples taken from the same patient on the same day) were removed from further analysis.

Resolving Inconsistencies

Inconsistent data points (n = 3) were identified (e.g., a data point that suggested a seropositive patient subsequently lost their antibody response). In these cases: samples were rerun, including sequential samples from the same patient from either side of the timepoint; alkaline phosphatase results from that sample were checked; and sample aliquots were resourced from stocks in South West London Pathology laboratories.

Results

Normalized ELISA Values

Normalized OD values for ELISA results are presented in Appendix Figure 1.

Analysis

A 1-way analysis of variance was conducted to compare the effect of race (white/nonwhite/unknown) on demographics and severity indices in patients. Results showed that antibody levels (mean NOD) across the 3 groups were unequal, $F(2,174) = 3.46$, $p = 0.03$ and post hoc comparisons using Tukey's test showed that antibody levels in whites were significantly different from nonwhites. There was no observed significant effect of race on other measured severity indices and demographics (Appendix Table 3).

Multiple Linear Regression (Independent Variable: Mean NOD)

Multiple linear regression was performed to determine the relationship between mean NOD and age, sex, peak CRP levels, number of concurrent conditions, presence or absence of respiratory symptoms, and race. Upon examination of regression β coefficients and associated t-statistic p values, there was a significant association found between higher age, higher peak CRP levels, nonwhite race, and higher NOD values. The p value of the F-statistic is 8.6×10^{-5} with an adjusted R-squared (R^2) of 0.10 (10% of the variance in mean NOD levels can be predicted by the 3 variables).

Our final model equation can be written as follows:

mean NOD = 0.38 + 0.005 × age + 0.001 × peak CRP level – 0.018 × race.

Race was coded as a dummy quantitative variable (0 = nonwhite race, 1 = white race).

Logistic Regression Model (Independent Variable: Seroconversion)

Logistic regression was performed to determine the relationship between seroconversion and age, sex, peak CRP levels, number of concurrent conditions, presence or absence of respiratory symptoms, and race. A significant association was found between higher age, higher peak CRP levels and seroconversion ($p = 0.001$ and $p = 0.035$ respectively).

Our final model equation can be written as follows:

Log odds of seroconversion = –1.37 + 0.051 × age + 0.004 × peak CRP level

Logistic Regression Model (Independent Variable: Severity/outcome)

Logistic regression was performed to determine the relationship between poor outcome (death and or ICU admission) and age, gender, peak CRP levels, number of concurrent conditions, presence or absence of respiratory symptoms, and race. A significant association was found between only higher peak CRP levels and poor outcome ($p = 1.1 \times 10^{-8}$).

Our final model equation can be written as follows:

Log odds of a poor outcome = –2.08 + 0.008 × peak CRP levels.

Appendix 1 Table 1. Expanded characteristics of patients with severe acute respiratory syndrome, United Kingdom, 2020

Characteristic	No. patients (%)
Race*	
White	60 (33.9)
British	38 (21.5)
Irish	1 (0.6)
Other	21 (11.9)
Nonwhite	61 (34.5)
Black or black British	
African	13 (7.3)
Caribbean	9 (5.1)
Other	5 (2.8)
Asian or Asian British	
Bangladeshi	1 (0.6)
Indian	6 (3.4)
Pakistani	2 (1.1)
Other	22 (12.4)
Mixed	3 (1.7)
Other/not known	56 (31.6)
Other	33 (18.6)
Not stated	23 (13.0)
Concurrent conditions	
None	47 (26.6)
Diabetes mellitus	52 (29.4)
Hypertension	75 (42.4)
Obesity (body mass index >30)†	44 (26.8)
Chronic kidney disease	27 (15.3)
Without dialysis	15 (8.5)
Hemodialysis	10 (5.6)
Renal transplant	2 (1.1)
Cancer	18 (10.2)
Asthma	21 (11.9)
Chronic obstructive pulmonary disease	6 (3.4)

*Race data are routinely collected by the National Health Service and categorised based on the 2001 UK Census classification.
†Height data unavailable for 13 patients.

Appendix 1 Table 2. Examination of demographics and severity indices in patients with severe acute respiratory syndrome 2, United Kingdom, 2020

Variable	F statistic	p value
Mean normalized optical density	3.46	0.03
Age	0.70	0.50
Gender	0.33	0.72
Body mass index	0.08	0.93
Concurrent conditions	1.26	0.29
Respiratory symptoms	0.72	0.49
Length of stay, d	1.38	0.26
Poor outcome (death and or intensive care unit)	0.99	0.38
Peak C-reactive protein levels	1.03	0.36

Appendix 1 Table 3. Laboratory values at diagnosis from symptomatic and asymptomatic patients with severe acute respiratory syndrome coronavirus 2, United Kingdom, 2020.

	Median (IQR), n = 177		
Test (reference range)	Symptomatic	Asymptomatic	p value*
C-reactive protein (0–5 mg/L)	97 (57.5–194), n = 113	56 (5.5–100.5), n = 21	<0.01
Nadir lymphocytes count (1.1–4.0 × 10^9/L)	0.9 (0.6–1.4), n = 113	1.0 (0.55–1.3), n = 21	0.76
Ferritin (30–400 μg/L)	1,134 (659.8–1,829), n = 40	222 (121–323), n = 2	0.04
Fibrinogen (1.6–4.8 g/L)	5.5 (4.3–6.7), n = 116	4.3 (2.9–6.25), n = 17	0.05
D-dimer (21–300 ng/mL)	705 (397–1,105), n = 59	703 (320–3,536), n = 5	0.91
Lactate dehydrogenase (0–250 U/L)	497 (282.3–600.3), n = 44	225 (210–703), n = 3	0.44

*2-tailed Mann-Whitney test.

Appendix 1 Table 4. Peak laboratory values in symptomatic and asymptomatic patients with severe acute respiratory syndrome coronavirus 2, United Kingdom, 2020

	Peak value Median (IQR)		
Test (reference range)	Symptomatic	Asymptomatic	p value*
C-reactive protein (0–5 mg/L)	255 (134.5–344.5), n = 142	104 (70.8–190.8), n = 34	<0.01
Nadir lymphocytes count (1.1–4.0 × 10^9/L)	0.5 (0.4–0.8), n = 143	0.65 (0.375–0.9), n = 34	0.46
Ferritin (30–400 μg/L)	1,363 (1,019–2,848), n = 79	624 (338–1,497), n = 10	0.02
Fibrinogen (1.6–4.8 g/L)	7.6 (5.6–9.5), n = 135	1.18 (1.18–1.18), n = 2	<0.01
D-dimer (21–300 ng/mL)	2,534 (693.8–5,754), n = 96	460 (293–1,726), n = 15	<0.01
Lactate dehydrogenase (0–250 U/L)	534 (363–707.5), n = 77	401 (202.5–740.5), n = 10	0.24

*2-tailed Mann-Whitney test.

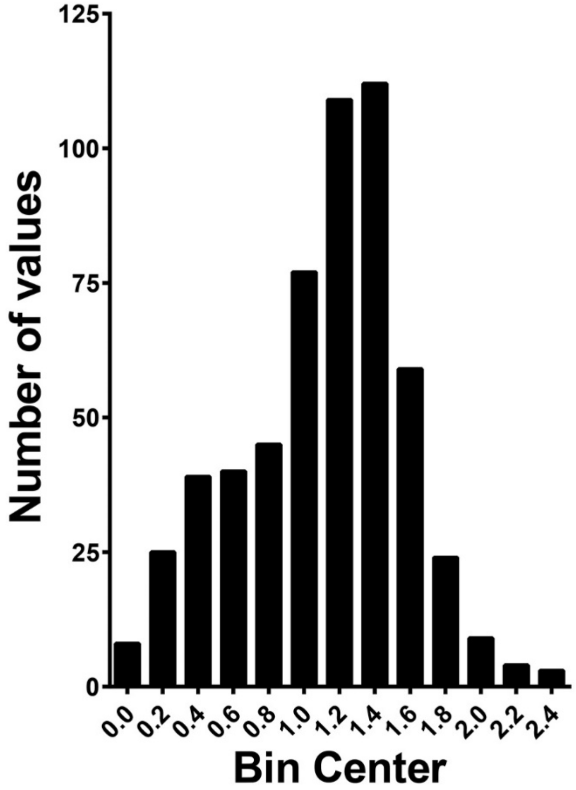

Appendix 1 Figure 1. Normalization of raw ELISA values of patients with severe acute respiratory syndrome coronavirus 2, United Kingdom, 2020.

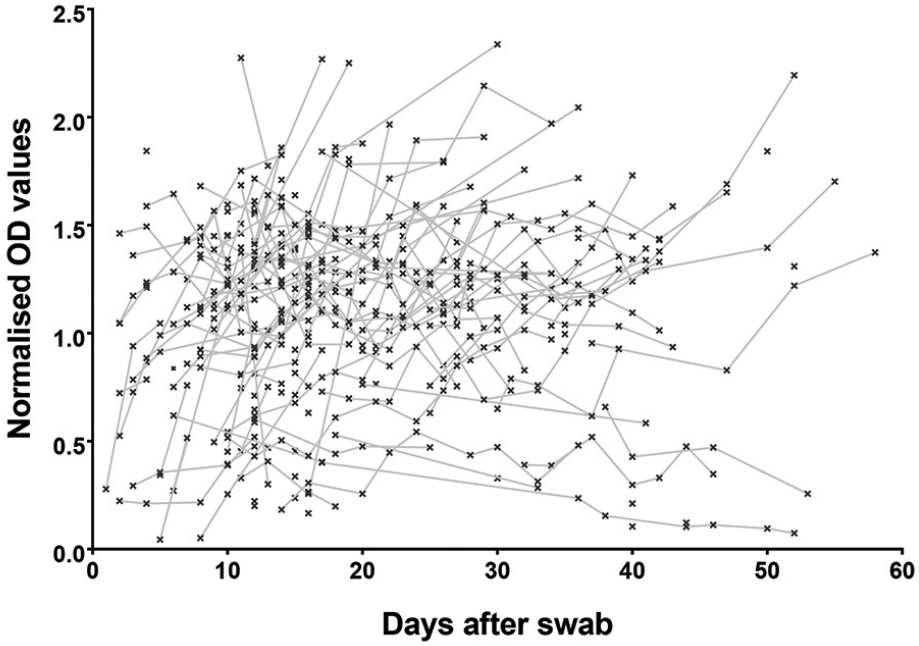

Appendix 1 Figure 2. NOD values by days after first positive PCR result for patients with severe acute respiratory syndrome 2 who had seroconverted at the time of first assay, United Kingdom, 2020. NOD, normalized optical density.

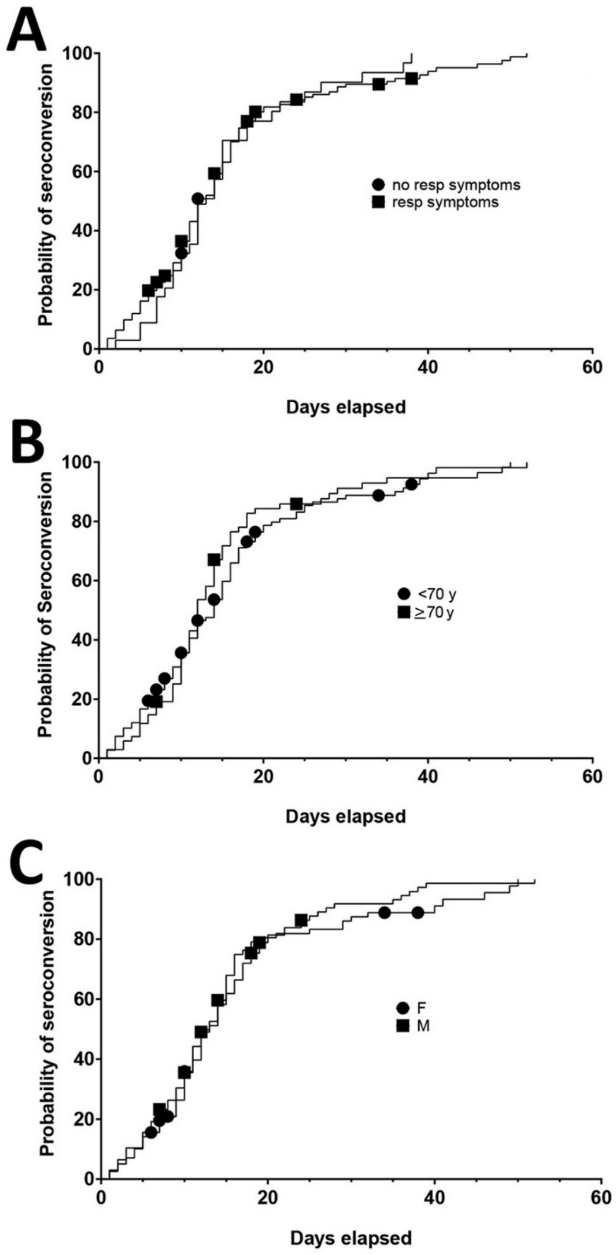

Appendix 1 Figure 3. Cumulative frequency plots for seroconversion of patients with severe acute respiratory syndrome coronavirus 2, United Kingdom, 2020. Probability of seroconversion is described in days after first positive PCR positive result. A) Patients with (filled squares) or without (filled circles) respiratory symptoms. Symbols indicate the last available seronegative sample for a patient subsequently right censored (i.e, no further data was available). B) Patients ≥70 years of age (filled squares) or <70 years of age (filled circles). Symbols indicate the last available seronegative sample for a patient subsequently right censored. C) Men (filled squares) or women (filled circles). Symbols indicate the last available seronegative sample for a patient subsequently right censored.

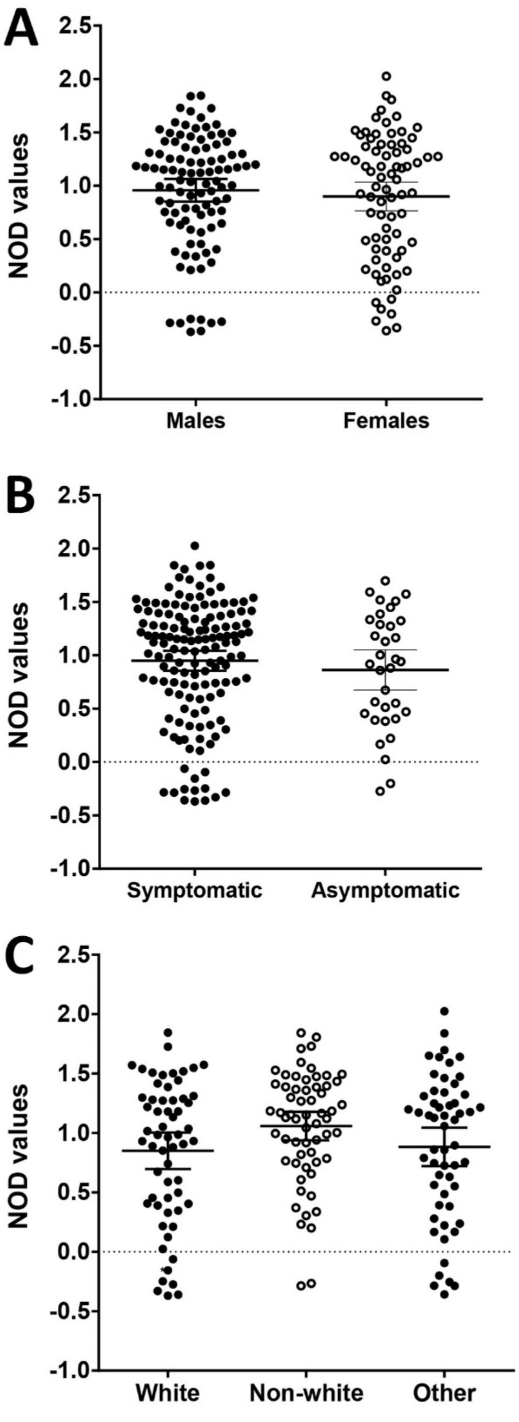

Appendix 1 Figure 4. NOD values compared by sex, symptoms, and race of patients with severe acute respiratory syndrome coronavirus 2, United Kingdom, 2020. A) Comparison of NOD values by sex (mean ± 95% CI; not significantly different); B) Comparison of NOD values by symptoms (mean ± 95% CI; not significantly different); C) Comparison of NOD values by race (mean ± 95% CI; $p = 0.04$, $F = 1.61$, df = 119; unpaired Student *t*-test between white and nonwhite). The third group is not known/other. NOD, normalized optical density.
